# Development of a New Limiting-Antigen Avidity Dot Immuno-Gold Filtration Assay for HIV-1 Incidence

**DOI:** 10.1371/journal.pone.0161183

**Published:** 2016-08-11

**Authors:** Zhiyun Gao, Hao Yan, Xia Feng, Lijin Wu, Maofeng Qiu, Wenge Xing, Guiyun Zhang, Zhi Zhang, Yan Jiang

**Affiliations:** 1 National HIV/HCV Reference Laboratory, National Center for AIDS/STD Control and Prevention, Chinese Center for Disease Control and Prevention, Beijing, China; 2 Department of Pathogenic Biology, Hebei Medical University, Shijiazhuang, China; 3 Zhejiang Provincial Center for Disease Control and Prevention, Hangzhou, China; 4 Beijing YouAn Hospital, Capital Medical University, Beijing, China; 5 Beijing KingHawk Pharmaceutical Co., Ltd., Beijing, China; New York Blood Center, UNITED STATES

## Abstract

Several laboratory assays on cross-sectional specimens for detecting recent HIV infections were developed, but these assays could not be applied in resource-limited and high HIV-incidence areas. This study describes the development of a rapid assay that can simultaneously detect the presence of HIV-1 antibodies of current and/or recent infection. The dot immuno-gold filtration assay (DIGFA) was used to detect recent infection on the principle of antibody avidity changes between recent and long-term infections. The dot immuno-gold silver staining filtration assay (DIGSSA) increases the sensitivity and accuracy of antibody detection by adding a silver staining step to the DIGFA. In the meantime the digital results were produced by the scanner for ambiguous specimens. Further, HIV-1 routine diagnostic antibody was detected simultaneously for improving practicability. The performance of the assays was then assessed through five serum panels with known serological statuses and seroconversion dates. The proportion of false recent infection (PFR) of the DIGSSA was obtained. Through the optimization of basic parameters for DIGSSA, six specimens were all classified correctly. DIGSSA demonstrated good repeatability and high sensitivity. The agreement of DIGSSA with the BED assay was 92.10% (κ = 0.65) and 95.36% with the LAg-Avidity assay (κ = 0.75). Moreover, the gray values of DIGSSA correlated well with BED ODn (R^2^ = 0.9397) and LAg-Avidity ODn (R^2^ = 0.9549). The PFR of DIGSSA was 2.73%, which was lower than that of the BED assay but higher than that of the LAg-Avidity assay. The DIGSSA can feasibly be applied to detect HIV infection and estimate HIV incidence.

## Introduction

Estimating human immunodeficiency virus (HIV) incidence is a vital component of monitoring the current HIV epidemic. This is important to understanding the HIV-1 transmission dynamics, identifying high-risk populations, and evaluating the effectiveness of prevention strategies [1]. Traditional methods for monitoring HIV-1 incidence depend on following a prospective cohort of individuals who are at risk of infection. With the development of laboratory techniques, several laboratory-based assays that distinguish between recent and long-term HIV-1 infection have been recommended to estimate HIV-1 incidence from cross-sectional samples [2]. These laboratory methods avoid limitations of prospective studies such as bias, logistics, and high costs [3–4]. Among these assays, the HIV-1 BED capture enzyme immunoassay (BED-CEIA) has been used in population surveillance globally [5–7]. However its accuracy has always been questionable due to high false recent classification which causes overestimations of HIV incidence [8] and the inconsistent mean duration of recent infection in different populations [9]. To increase the accuracy of HIV incidence estimates, a new limiting antigen avidity enzyme immunoassay (LAg-Avidity EIA) dependent on antibody avidity increasing gradually over time was developed [10–12]. The assay was expected to be more robust in distinguishing recent from long-term infection since antibody avidity is a property of maturing antibodies [12]. Recent studies demonstrated that the new assay has a significantly lower false recent rate than BED-CEIA [13–15].

These assays based on EIA are easy to perform, but are time-consuming, require special laboratory equipment, and highly trained staff. For usage in resource-poor laboratories, we focused on developing rapid HIV incidence tests. Commercially available rapid HIV tests were modified as less sensitive assays to identify recent HIV infection [16]. Although these modified assays can be used to estimate HIV-1 incidence, complicated steps for dilution and subtype B-derived antigen limit their practical application [17–18]. The recently developed lateral flow assay detects recent HIV-1 infection by measuring antibody avidity with multi-subtype-recombinant proteins. The results are consistent with BED assay in 95.1%. However, the low volume of specimen used (1μl of serum) and a dilution factor of 200 increases the risks of experimental error [9]. Also, these assays are single-use and have poor sensitivity. It is therefore necessary to develop a rapid test for HIV-1 incidence that is easy-to-use, and has high sensitivity and accuracy.

Dot immuno-gold filtration assay (DIGFA) is a rapid membrane-based immunodiagnostic technique. Compared with EIA, this assay is more suitable for on-site testing due to its rapid, convenient, economical, and visual characteristics [19]. However, DIGFA has the same advantages with other rapid detection techniques, such as immune-chromatography. In 1995, silver staining was added to gold-labeled immune technology and became immune-gold-silver stained [20]. Due to the signaling cascade of silver staining, the detection sensitivity of DIGFA was improved. Immune-gold-silver staining technology became widely used thereafter [21].

Considering that HIV-1 positive status must be confirmed before detections of recent infection with commercial incidence assays and the deficiency of the rapid assays, in this study, we modified the dot immuno-gold silver staining filtration assay (DIGSSA) with a method which was based on the principle of limiting antigen avidity measurement, and developed a rapid HIV test for detection of recent HIV-1 infection through combination with a immune-gold silver staining filtration assay. Moreover HIV-1 antibodies were detected with high practicability and convenience.

## Materials and Methods

### Specimens

Five serum panels were used to develop and assess the DIGSSA (Table 1). Panel 2 was purchased from China Food and Drug Administration (CFDA), while others were obtained from the National HIV/HCV Reference Laboratory of the Chinese Center for Disease Control and Prevention. According to the national guidelines for HIV/AIDS detection, all specimens were tested for HIV serological status through the standard enzyme-linked immunosorbent screening assay and Western blot confirmatory test [22].

**Table 1:** Specimen panels for development, optimization, and characterization of DIGSSA.

Specimen set	NO.	Negative	Positive		Recent determination method	Purpose
			RT	LT		
Panel 1	6	1	2	3	Seroconversion date	Assay optimization
Panel 2	40	20	20 ^a^		-	Assessment of Assay
Panel 3	185	109	21^b^	55^b^	BED-CEIA and LAg-Avidity EIA incidence assay	Assessment of Assay
Panel 4	250	0	43 ^c^	157^d^	Seroconversion date	Detection of Recent infection
Panel 5	256	0	0	256	Seroconversion date	Detection of PFR

DIGSSA: dot immune-gold filtration assay. RT: Recent infection; LT: Long-term infection. PFR: the Proportion of false recent infection. BED-CEIA: BED capture enzyme immunoassay. LAg-Avidity EIA, the limiting antigen avidity enzyme immunoassay.

^a^: including 18 HIV-1 antibody-positive samples, 2 HIV-2 antibody-positive samples

^b^: 76 samples classified according to LAg-Avidity EIA, while RT/LT = 28/48 according to the BED assay

^c^: seroconversion date<180d

^d^: seroconversion date>365d.

Panel 1 included six control samples, which was used to optimize the test parameters and develop the performance traits of DIGSSA. In panel 1, HIV-1 positive specimens were categorized as recent or long-term according to the known seroconversion dates. Other four panels were used for assessing the performance of DIGSSA. Panel 2 was a national reference panel for anti-HIV on the rapid colloidal gold labeling test, whose information of specimens provided by CFDA. Panel 3 was collected from different provinces of China in 2011, including 76 HIV-1 antibody-positive and 109 antibody-negative samples. Since these HIV-1 antibodies lack seroconversion dates, these samples were classified according to the results of LAg-Avidity EIA and the BED assay. Panel 4 was a cohort of 250 specimens including 62 treatment-naïve people living with HIV/AIDS (PLHIV), whose infection dates were estimated from dates of their last HIV-negative and first HIV-positive tests. Panel 5 was collected from individuals who were infected more than one year ago and were not enrolled in antiretroviral therapy. These anti-HIV positive specimens were classified using commercial incidence assays or seroconversion date.

Ethical approval to conduct the study was granted by the Institutional Review Board of the National Center of AIDS/STD Control and Prevention, Chinese Center for Disease Control and Prevention. Written informed consent was obtained from all participants for their clinical records to be used in this study.

### HIV antigens and antibody reagents

Probe 1 (the mouse anti-human IgG monoclonal antibody) and the colloidal gold conjugated goat-anti-human IgG were purchased from Shenzhen FAPON Biology Co., Ltd. Probes 2 (HIV-1 gp41 recombination protein) and 3 (HIV-1 multisubtype gp41 recombination protein, rIDR-M) were provided by Beijing King Hawk Pharmaceutical Co., Ltd.

### Preparation of DIGFA

**Fig 1:**
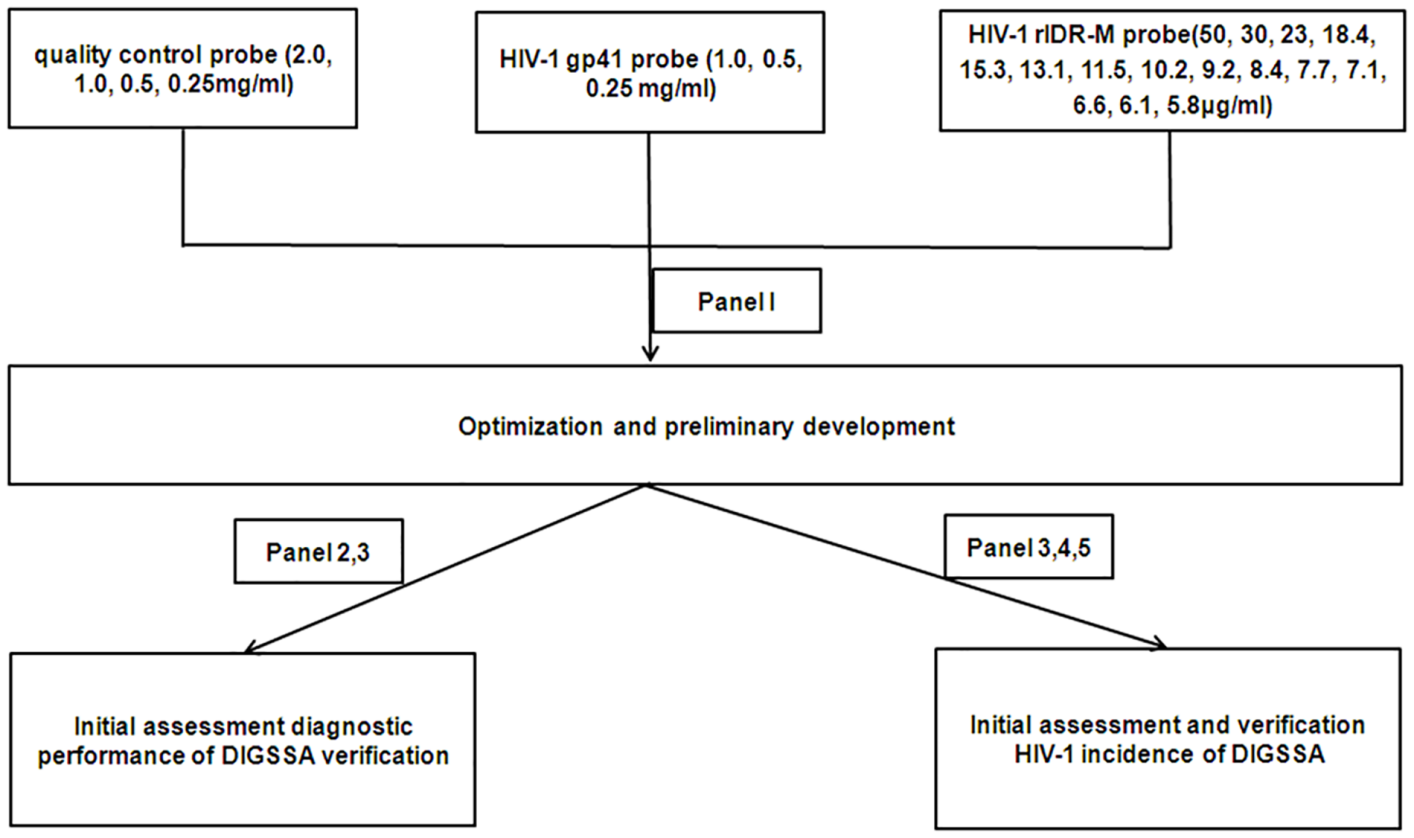
The flow chart for optimizing DIFGA.

Three different probes were used to design this assay. Probe 1 was used for quality control of assay performance, while probes 2 and 3 were used respectively to detect diagnostic antibodies and distinguish between recent and long-term HIV-1 infection. For optimizing the assay, the three probes were reconstituted in different concentrations and tested in panel 1. The optimized assay was then assessed and verified. The optimized flow chart and the serum panels used are shown in Fig 1.

**Fig 2:**
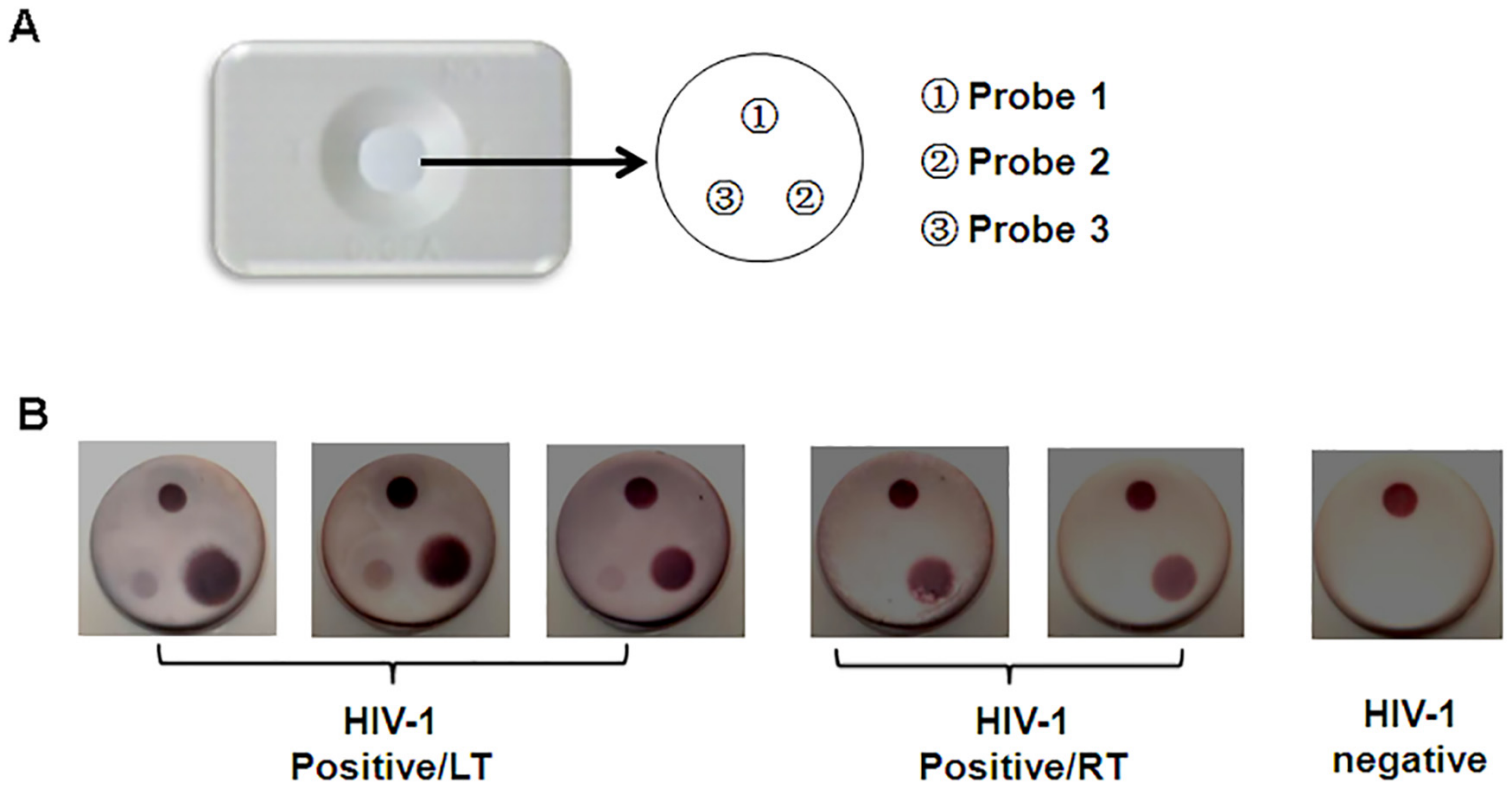
The diagram of sampling location and results of DIGFA. (A) the diagram of sampling location (B) the result photos of panel 1. three long-term infections (>1year) (left); two recent infections (< 4 months) (middle); one HIV negative (right).

The immunofiltration device used in this study was prepared as described previously [23]. After assembling the DIGFA device, 0.5μl of each probe was dripped onto the nitrocellulose filter membrane (NCM) forming a small dot and then dried at 37°C for five hours. The finished device was stored at 4°C (Fig 2A). The protein coating buffer consisted of 2% isopropyl alcohol in 0.01M phosphate buffer solution (PBS, pH 7.4).

### DIGFA procedure

Before the optimized DIGFA was performed, samples were diluted twice with blocking buffer (25% fetal bovine serum in 0.01M PBS, pH 7.4). The reaction was started by dripping three drops (about 100μl) washing buffer (1 ‰Tween-20 in 0.01M PBS, pH 7.4) to saturate the membrane. Blocking buffer was used on the test NCM (pore size 0.45μm) to eliminate non-specific binding. After the blocking liquid was totally absorbed, 40μl diluted specimen was added. Next, the test membrane was washed with six drops (about 200μl) of washing buffer. Finally, 40μl of colloidal gold conjugated antibody was dripped on the test hole in the same manner as the washing membrane procedure as described above. Each reagent was added after the previous fluid was completely absorbed. The results were observed and recorded immediately. The results were interpreted as follows: there should always be a visible dot in the control position, otherwise the assay was invalid and the specimen must be re-tested. If only probe 1 appeared, the sample was considered negative; if probes 2 and 3 appeared, the specimen was classified as long-term HIV-1 infection. If only probe 2 appeared, the specimen was classified as recent HIV-1 infection. If only probe 3 appeared the assay was considered invalid.

### DIGSSA procedure

To enhance the detection sensitivity, a silver staining enhancement reagent was used to amplify the signal of colloidal gold. When finishing the DIFGA test, three drops (about 100μl) of deionized water were dropped into the test hole to remove impurities. Then 50μl of silver enhancer with equal volume of solution A (aqueous solution containing 0.02M silver acetate) and solution B (citrate buffer containing 0.1M hydroquinone) were added. After the reaction was under a black thick paper for a certain time, deionized water was added to end the reaction. The effect of silver staining is directly related to the volume of reagents and staining time. In order to standardize the silver staining procedure, different staining times (2, 3, 4, 5, and 6 minutes) and reagent volumes (A+B:15+15μl, 25+25μl, 35+35μl) were tested. To eliminate measurement variability of the naked eye, Image J software (National Institutes of Health) was used to reflect the test results by quantifying the brightness of the probe signal. To minimize the sample and operation bias, we ruled that the probe gray value was equal to the foreground value minus background value.

As results were digitized, the corresponding interpretation of sample results needed be redefined. The cutoff value was used to determine whether the sample is positive or negative, especially for those samples with a weak signal. Therefore, cutoff values of every tested probe needed to be determined. For probe 2, two hundred negative samples were tested and test results were scanned and used to calculate the cutoff value. The cutoff value of probe 2 was the sum of the mean gray value and two standard deviations (SD). The cutoff value of probe 3 was difficult to determine because a large number of samples could not be obtained where infection duration was close to the mean duration of recent infection. Therefore, the cutoff value was determined under the condition that both the optimized probe 3 concentration and the gray value of the weakest dot were positive in at least two of three experimenters.

### Repeatability of DIGSSA

Three samples (1 long-term, 1 recent, 1 negative) were selected from panel 1 for the repeatability of the rapid assay. These specimens were tested five times with the same batch of DIGSSA for inter-assay variations or with three batches of DIGSSA for intra-assay variations. The blank control was set in every test. The gray values of all probes were collected and analyzed.

### HIV-1 BED incidence assay and HIV-1 LAg-Avidity assay

The BED-CEIA and the LAg-Avidity EIA are two kinds of mature methods for estimating HIV-1 incidence. The two assays were performed as previously described (BED-CEIA, Calypte Biomedical Corp, Portland, OR; LAg-Avidity EIA, Sedia Biosciences Corp, Portland, OR) [12,24]. The samples were classified as recent or long-term infections according to the respective threshold values. The results of recent samples tested by BED-CEIA suggested the individuals acquired HIV infection within a mean time period of 168 days [25], while those by LAg-Avidity EIA suggested that individuals acquired HIV infection within a mean time period of 130 days [26].

### Proportion of false recent infection (PFR) of DIGSSA

To obtain PFR of DIGSSA, samples from panel 5 were detected by BED-CEIA, the LAg-Avidity EIA and DIGSSA. The data of DIGSSA were compared to other two methods to assess the established assay.

### Data analysis

All data were stored in a Microsoft Excel database. Statistical Program for Social Sciences (SPSS, version 17.0) was used for all statistical analyses. Agreement between DIGSSA and commercial EIAs was analyzed by calculating the kappa statistics, PFR was assessed by chi-square test.

## Results

### DIGFA optimization

Basic parameters of the assay were determined according to the DIGSSA protocol. When probes 1 and 2 were at a concentration of 0.5mg/ml, probe 3 at 15.3μg/ml, through 25μl volume of solution A and B staining for 3min, six samples of panel 1 were all classified correctly (Fig 2B). Three long-term HIV-1 infection specimens showed three dots and two recent HIV-1 specimens displayed two dots with no dot in the probe 3 position. In addition, one negative specimen had one dot at control position.

### Cutoff value of probe 2 and 3

The cutoff value of probe 2 was determined to be 4.08 (mean+2SD = 1.32+2×1.38) based on the analysis of gray values of 200 negative samples. When the gray value was greater than or equal to 4.08, the sample was considered positive; when the gray value was less than 4.08, the sample was considered negative. When the gray value of probe 3 was between 3 and 4, the weakest dot could be recognized by at least two out of three laboratory technicians in sufficient light. Therefore, the cutoff value of probe 3 was determined to be 3.50. If the gray value was less than or equal to 3.50, the sample was classified as that of a recently infected patient, otherwise it was considered to be from a long-term infection.

### Repeatability of DIGSSA

The samples were tested five times by the same batch or three batches of DIGSSA. Scanned results show that the coefficient of variation of intra-assay and inter-assay was less than 15% (Table 2).

**Table 2:** Mean Gray values with standard deviation (SD) of three probes tested with the same batch and three batches of DIGSSA.

Probe	Sample	Inter-assay		Intra-assay	
		Mean±SD	CV (%)	Mean±SD	CV (%)
Probe 1	LT	39.60±2.97	7.49	35.33±5.13	14.52
	RT	43.40±2.07	4.78	38.33±3.51	9.16
	Negative	43.00±2.55	5.93	36.00±4.58	12.73
	Blank	1.40±0.89	63.89	1.67±0.58	34.64
Probe 2	LT	47.20±3.11	6.60	49.67±1.53	3.08
	RT	34.40±2.61	7.58	34.33±2.08	6.06
	Negative	2.20±0.84	38.03	2.00±1.00	50.00
	Blank	0.40±0.55	136.93	0.00±0.00	-
Probe 3	LT	9.80±1.30	13.30	10.67±1.53	14.32
	RT	2.40±0.89	37.27	2.67±0.58	21.65
	Negative	1.40±0.55	39.12	1.67±0.58	34.64
	Blank	0.40±0.55	136.93	0.00±0.00	-

DIGSSA: dot immune-gold filtration assay. SD: standard deviation. CV, coefficient of variation. LT: long-term infection. RT: recent infection.

### Diagnostic Performance of DIGSSA

The visual classifications of panels 2 and 3 detected by DIGSSA showed 224 of 225 specimens agreed with the criteria of the national reference panel and the results of western blot, and a negative specimen was misclassified as positive. The overall sensitivity and specificity of DIGSSA were 100% and 99%, respectively.

### Performance of DIGSSA for distinguishing HIV-1 recent and long-term infection

**Fig 3:**
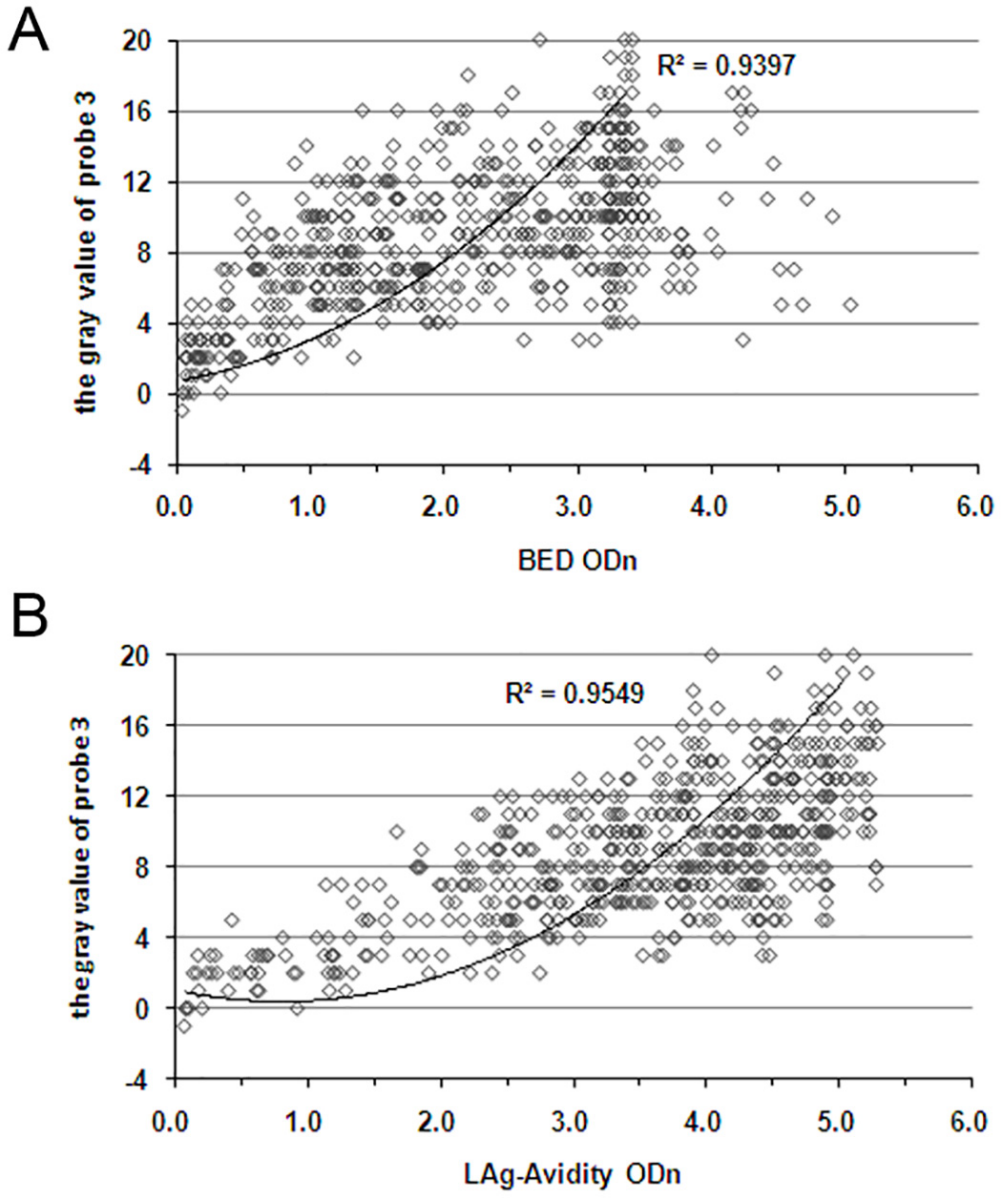
Concordance between the gray value of DIGSSA and BED-CEIA ODn or LAg-Avidity ODn. (A) concordance between the gray value of DIGSSA and BED-CEIA ODn, the horizontal arrows corresponds to a probe 3 cutoff of 3.50 and the vertical arrows corresponds to a BED ODn cutoff of 0.8. (B) concordance between the gray value of DIGSSA and LAg-Avidity ODn, the horizontal arrows corresponds to a probe 3 cutoff of 3.50 and the vertical arrows corresponds to a LAg-Avidity cutoff ODn of 1.5.

A total of 582 specimens were detected by DIGSSA, BED-CEIA and LAg-Avidity EIA. Results are shown in Tables 3 and 4. The details were shown in S1 Table. The overall agreement between DIGSSA and BED-CEIA was 92.10% (κ = 0.65, 95% CI = 0.55–0.74), while that between DIGSSA and LAg-Avidity EIA was 95.36% (κ = 0.75, 95% = CI 0.65–0.84). The concordance between the gray values of DIGSSA and the normalized optical density values (ODn) of the other two methods was further analyzed. Results were shown in Fig 3A and 3B. The gray value of DIGSSA had a high correlation with BED ODn and LAg-Avidity ODn, and the corresponding R^2^ were 0.940 and 0.955, respectively.

**Table 3:** Comparisons between DIGSSA and BED-CEIA for classifying recent or long-term infections.

BED-CEIA	DIGSSA		Total
	Recent	Long-term	
Recent	51	38	89
Long-term	8	485	493
Total	59	523	582

κ = 0.65 (95% CI = 0.55–0.74)

DIGSSA: dot immune-gold filtration assay. BED-CEIA: BED capture enzyme immunoassay. CI: confidence interval.

**Table 4:** Comparisons between DIGSSA and LAg-Avidity EIA for classifying recent or long-term infections.

LAg-Avidity EIA	DIGSSA		Total
	Recent	Long-term	
Recent	44	12	56
Long-term	15	511	526
Total	59	523	582

κ = 0.75 (95% CI = 0.65–0.84)

DIGSSA: dot immune-gold filtration assay. LAg-Avidity EIA: the limiting antigen avidity enzyme immunoassay. CI: confidence interval.

### The Proportion of false recent infections of DIGSSA

The DIGSSA misclassified 7 of 256 individuals who were actually infected more than one year as recent infections. The PFR was 2.73% (95%CI 0.73–4.73%) in panel 5. In comparison, the PFR with LAg-Avidity EIA was 0.39% (95%CI 0.00–1.15%) with 1 of the 256 specimens being misclassified as recent, while the PFR with BED-CEIA were 3.91% (95%CI 1.54–6.28%) with 10 individuals as recent infection.

Further chi-square analysis showed there was no significant difference in PFR between DIGSSA and BED-CEIA (*χ*^2^ = 0.55, *P* > 0.05). Similarity exists between DIGSSA and LAg-Avidity (*χ*^2^ = 3.17, *P* > 0.05). However, there is a significant difference between BED-CEIA and LAg-Avidity EIA (*χ*^2^ = 7.53, *P* < 0.01).

**Fig 4:**
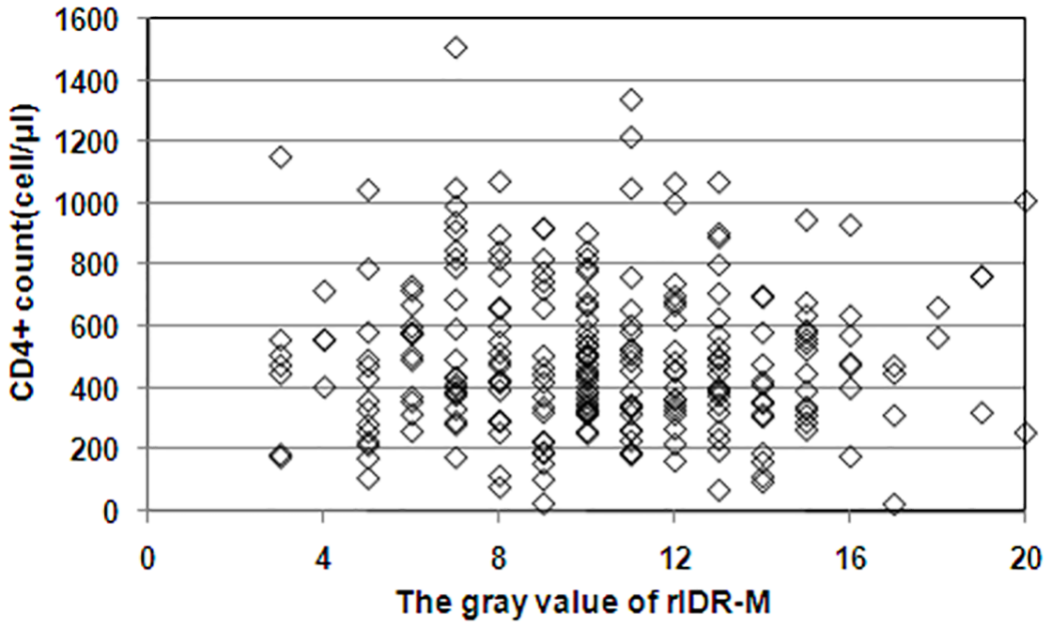
Concordance between CD4^+^ count and the gray value of DIGSSA.

CD4^+^ cell counts were available for 250 specimens, where 226 specimens had over 200 cells per microliter, 22 specimens between 50 and 200 cells per microliter, and two specimens less than 50 cells per microliter. Both BED-CEIA and DIGSSA misclassified two out of 22 specimens respectively, while the LAg-Avidity EIA made no misclassification. All three assays classified the specimens with CD4^+^ cells counts less than 50 cells per microliter correctly. The correlation between CD4^+^ cell count and the gray value of DIGSSA was analyzed using long-term infected specimens (Fig 4). The details were shown in S2 Table.

## Discussion

In this study, we describe a rapid approach to using an immune-gold silver staining filtration assay for detecting recent and long-term HIV-1 infection simultaneously. We used two different antigens as the detecting incidence probe and diagnostic probe. The rIDR-M protein was a multisubtype gp41 recombinant antigen and had high reaction with HIV-1 antibodies in sera from different subtypes [10]. So it was selected as detecting recent HIV probe. To enhance the practicality of this assay, a HIV diagnostic probe was added. A prior study shows that the sensitivity (97.5%) of EIA using rIDR-M as routine diagnostic probe was not suitable [10]. The common HIV-1 gp41 recombinant antigen was selected, which was applied in Chinese commercial rapid HIV testing reagents. It was proven that the reagents were highly sensitive in assessing the performance of Chinese HIV diagnostic reagents [27–29]. In our assessment, the sensitivity of this antigen was 100%. The two antigens were coated in NCM simultaneously in design.

To distinguish recent from long-term infections, a principle of LAg-Avidity EIA was applied; limited amount of antigen was coated and dissociation buffers were used in the subsequent procedure. Our early trials indicated that when dissociation buffers were added, the control signal declined while the non-specific response increased. A possible explanation for this is that the biological characteristics of NCM can be damaged by dissociation buffers. To achieve the goal of identifying serum incidence status, the dissociation stage was eliminated and the amount of coated antigen was further reduced in this assay. A similar procedure was reported in the research of Wei et al. [10]. Therefore, the amount of coated antigen became the crux of this assay. The final probe concentration was determined through serial dilutions with small differences reacting with those specimens, whose duration of infection are close to the window period and BED ODn or LAg-Avidity ODn are near the corresponding cutoff. The preliminary verification showed it could correctly distinguish between recent and long-term infections.

Problems may arise when smaller concentrations of the coated antigen are used. Most obviously, the sensitivity of the assay may decrease. To solve this problem, a silver staining step was added to amplify the detection signal. As a result, the dot became more visible. The results of some samples were still ambiguous and difficult to determine by the naked eye. A common scanner was introduced to detect the gray value of testing dots. The cutoff value was determined by two samples whose duration of infection are close to the mean duration of recency of BED-CEIA and LAg-Avidity EIA. Though the sample size was small, our data validation experiment showed 3.5 was a feasible cutoff value for the rIDR-M probe. Moreover, the repeatability of the assay was not affected by a smaller concentration of coated antigen.

To assess the performance of the DIGSSA diagnostic probe, the national anti-HIV reference panel was tested in our study. The data showed the DIGSSA probe has high sensitivity (100%) and specificity (99%), and reported values identical to those reported in the national performance evaluation of commercial HIV screening tests available in the Chinese market. The detection results of another panel was also similar with the WB confirmatory tests. However, further assessment is needed due to the small sample size used in this study.

We compared the results of DIGSSA with the commercial LAg-Avidity EIA and BED-CEIA and showed 95.36% (κ = 0.75) and 92.10% (κ = 0.65) agreement, respectively. Moreover, there was a high correlation between DIGSSA gray value and two other EIA ODn (R^2^ = 0.955, 0.940). Better agreement was observed between DIGSSA and LAg-Avidity EIA. One possible reason is the similarity of principles for DIGSSA and LAg-Avidity EIA. The higher PFR of BED-CEIA [18] is another reason, though nearly half of the discordant specimens (19/46) had BED ODn values near the threshold. The PFR can vary in based on population, HIV subtypes, and the geographic region. However, in the same sample group, when the method has a lower PFR, the corresponding misclassification probability of long-term samples is reduced. In this study, the PFR of DIGSSA was 2.73%, while the PFR of LAg-Avidity EIA and BED-CEIA 0.39% and 3.91% respectively. The statistical analysis of the PFR among three assays indicated DIGSSA may have lower PFR than BED-CEIA with larger sample sizes. In addition, although the DIGSSA PFR (2.73%) had not reached the WHO-recommended level of 2% [30], this PFR can meet on-site screening demands which exclude most long-term samples. If the effective strategies match with DIGSSA, the costs will be reduced and the testing efficiency will be greatly improved.

Current laboratory assays may misclassify patients who are infected long-term but with CD4+ cell counts less than 200 cell per microliter or who started antiviral treatment (ART) during acute infections. Those kind of samples should be excluded when calculating local HIV-1 incidence. In our study, we included 256 samples in panel 5 in order to evaluate the PFR of DIGSSA. Those samples were all long-term infected but not currently on ART treatment, and 24 of them had CD4+ cell counts less than 200 cell per microliter. The small sample numbers limited the evaluation of the relationship between CD4+ cell counts and PFR. However, the correlation between CD4+ cell count and the DIGSSA gray value indicated CD4+ cell count had little influence on DIGSSA in detecting the samples who have not initiated ART treatment. In addition, viral load (VL) played an important role in PFR of BED-CEIA [31]. Unfortunately, VL information of the panel 5 specimens were lacked, its influence on DIGSSA could not be evaluated in our study.

Our new approach shows several advantages. First, it provides reliable results in 3 to 5 mins, without requiring sophisticated equipment and well-skilled laboratory staff. Second, double dilution of specimens may reduce misoperation associated with multiple dilution in other methods. Third, detections of recent infection are traditionally performed after confirming HIV-1 positive status. However, the DIGSSA design simplifies the procedure by detecting recent or long-term infection in a single device. Therefore, this approach establishes a cost-effective way for measuring prevalence and incidence of HIV in a population.

In conclusion, our data demonstrates that DIGSSA can be used for initial screening of HIV infection and detecting recent infections. For further application of DIGSSA, more work is required to verify and assess the assay using a larger sample size, and establishing the mean duration and simplifying the usage of the assay.

## Supporting Information

S1 Table Total results of Panel 3, 4 and 5 detected by BED-CEIA, LAg-Avidity EIA and DIGSSA. (DOCX)

S2 Table BED-CEIA ODn, LAg-Avidity ODn, the gray values and CD4^+^ count of specimens from panel 5. (DOCX)
