# Supplementary material for: Development of a New Limiting-Antigen Avidity Dot Immuno-Gold Filtration Assay for HIV-1 Incidence
Source: PLoS One. 2016 Aug 11;11(8):e0161183. doi: 10.1371/journal.pone.0161183 (PMC4981313; doi:10.1371/journal.pone.0161183)
Supplement: S1 Table — (DOCX) [file pone.0161183.s001.docx]

**S1 Table. Total results of Panel 3, 4 and 5 detected by BED-CEIA, LAg-Avidity EIA and DIGSSA**

| **NO.** | **ODn**  **LAg-Avidity EIA** | **ODn**  **BED-CEIA** | **Gray Value**  **DIGSSA** |
| --- | --- | --- | --- |
| 1 | 0.073 | 0.039 | -1 |
| 2 | 0.087 | 0.046 | 0 |
| 3 | 0.107 | 0.084 | 0 |
| 4 | 0.085 | 0.047 | 0 |
| 5 | 0.921 | 0.329 | 0 |
| 6 | 0.617 | 0.072 | 1 |
| 7 | 1.286 | 0.228 | 1 |
| 8 | 0.635 | 0.210 | 1 |
| 9 | 1.164 | 0.403 | 1 |
| 10 | 0.404 | 0.110 | 1 |
| 11 | 0.279 | 0.065 | 2 |
| 12 | 0.169 | 0.077 | 2 |
| 13 | 0.892 | 0.240 | 2 |
| 14 | 1.211 | 0.420 | 2 |
| 15 | 0.916 | 0.132 | 2 |
| 16 | 0.483 | 0.139 | 2 |
| 17 | 1.339 | 0.462 | 2 |
| 18 | 2.387 | 0.716 | 2 |
| 19 | 2.222 | 0.472 | 2 |
| 20 | 1.910 | 0.930 | 2 |
| 21 | 0.449 | 0.184 | 2 |
| 22 | 2.745 | 1.322 | 2 |
| 23 | 0.140 | 0.063 | 2 |
| 24 | 0.689 | 0.105 | 3 |
| 25 | 0.657 | 0.181 | 3 |
| 26 | 0.268 | 0.065 | 3 |
| 27 | 0.305 | 0.100 | 3 |
| 28 | 2.575 | 0.710 | 3 |
| 29 | 1.556 | 0.298 | 3 |
| 30 | 3.668 | 3.008 | 3 |
| 31 | 3.519 | 4.234 | 3 |
| 32 | 0.703 | 0.173 | 3 |
| 33 | 1.183 | 0.228 | 3 |
| 34 | 1.452 | 0.357 | 3 |
| 35 | 2.424 | 0.813 | 4 |
| 36 | 2.556 | 0.900 | 4 |
| 37 | 0.812 | 0.160 | 4 |
| 38 | 1.316 | 0.074 | 4 |
| 39 | 1.597 | 0.480 | 4 |
| 40 | 3.042 | 1.235 | 4 |
| 41 | 2.824 | 1.947 | 4 |
| 42 | 3.751 | 3.265 | 4 |
| 43 | 3.639 | 3.405 | 4 |
| 44 | 2.806 | 0.717 | 4 |
| 45 | 2.875 | 1.539 | 4 |
| 46 | 4.166 | 2.047 | 4 |
| 47 | 4.075 | 3.311 | 4 |
| 48 | 1.068 | 0.282 | 4 |
| 49 | 3.943 | 1.960 | 5 |
| 50 | 3.102 | 1.053 | 5 |
| 51 | 2.799 | 1.696 | 5 |
| 52 | 1.897 | 0.614 | 5 |
| 53 | 3.006 | 2.428 | 5 |
| 54 | 1.455 | 0.209 | 5 |
| 55 | 4.380 | 4.521 | 5 |
| 56 | 4.399 | 4.680 | 5 |
| 57 | 2.170 | 1.323 | 5 |
| 58 | 2.518 | 1.353 | 5 |
| 59 | 4.015 | 3.239 | 5 |
| 60 | 1.413 | 0.376 | 5 |
| 61 | 2.305 | 1.238 | 5 |
| 62 | 3.067 | 1.086 | 5 |
| 63 | 2.490 | 1.134 | 5 |
| 64 | 2.801 | 1.199 | 5 |
| 65 | 2.530 | 1.023 | 5 |
| 66 | 2.357 | 1.298 | 5 |
| 67 | 2.884 | 0.753 | 5 |
| 68 | 4.902 | 5.039 | 5 |
| 69 | 4.054 | 1.857 | 5 |
| 70 | 3.858 | 2.080 | 5 |
| 71 | 1.456 | 0.380 | 5 |
| 72 | 1.629 | 0.700 | 6 |
| 73 | 2.404 | 0.893 | 6 |
| 74 | 3.142 | 1.216 | 6 |
| 75 | 2.898 | 1.358 | 6 |
| 76 | 2.558 | 2.075 | 6 |
| 77 | 2.051 | 1.060 | 6 |
| 78 | 4.195 | 3.833 | 6 |
| 79 | 2.540 | 1.586 | 6 |
| 80 | 3.863 | 3.761 | 6 |
| 81 | 3.545 | 1.611 | 6 |
| 82 | 4.099 | 2.695 | 6 |
| 83 | 3.741 | 1.140 | 6 |
| 84 | 3.518 | 1.972 | 6 |
| 85 | 2.345 | 0.855 | 6 |
| 86 | 3.350 | 2.873 | 6 |
| 87 | 2.948 | 1.369 | 6 |
| 88 | 3.468 | 1.494 | 6 |
| 89 | 3.143 | 2.870 | 6 |
| 90 | 2.107 | 0.743 | 6 |
| 91 | 3.191 | 1.271 | 6 |
| 92 | 4.006 | 1.542 | 6 |
| 93 | 3.744 | 1.860 | 6 |
| 94 | 4.444 | 1.654 | 6 |
| 95 | 3.443 | 2.333 | 6 |
| 96 | 3.221 | 3.677 | 6 |
| 97 | 2.693 | 0.829 | 6 |
| 98 | 2.678 | 1.325 | 6 |
| 99 | 2.824 | 1.043 | 6 |
| 100 | 3.836 | 1.798 | 6 |
| 101 | 4.238 | 2.103 | 6 |
| 102 | 3.389 | 1.042 | 6 |
| 103 | 1.343 | 0.375 | 6 |
| 104 | 3.631 | 1.373 | 7 |
| 105 | 2.425 | 1.132 | 7 |
| 106 | 2.865 | 1.480 | 7 |
| 107 | 2.703 | 0.913 | 7 |
| 108 | 2.823 | 1.063 | 7 |
| 109 | 1.409 | 0.598 | 7 |
| 110 | 2.290 | 0.435 | 7 |
| 111 | 2.189 | 0.870 | 7 |
| 112 | 3.194 | 1.139 | 7 |
| 113 | 2.834 | 0.861 | 7 |
| 114 | 3.828 | 3.011 | 7 |
| 115 | 3.550 | 2.675 | 7 |
| 116 | 3.904 | 3.282 | 7 |
| 117 | 2.764 | 1.615 | 7 |
| 118 | 3.142 | 1.261 | 7 |
| 119 | 3.110 | 2.290 | 7 |
| 120 | 2.014 | 0.628 | 7 |
| 121 | 2.513 | 1.822 | 7 |
| 122 | 2.602 | 1.249 | 7 |
| 123 | 4.388 | 4.616 | 7 |
| 124 | 3.036 | 1.862 | 7 |
| 125 | 4.364 | 2.371 | 7 |
| 126 | 3.968 | 3.097 | 7 |
| 127 | 3.842 | 1.850 | 7 |
| 128 | 4.292 | 3.710 | 7 |
| 129 | 4.334 | 3.847 | 7 |
| 130 | 3.372 | 1.241 | 7 |
| 131 | 3.181 | 0.974 | 7 |
| 132 | 3.361 | 1.324 | 7 |
| 133 | 4.881 | 4.510 | 7 |
| 134 | 3.199 | 0.581 | 7 |
| 135 | 4.279 | 1.661 | 7 |
| 136 | 2.252 | 0.805 | 7 |
| 137 | 4.030 | 1.485 | 7 |
| 138 | 3.012 | 0.883 | 8 |
| 139 | 3.978 | 3.755 | 8 |
| 140 | 3.869 | 4.048 | 8 |
| 141 | 3.264 | 2.231 | 8 |
| 142 | 3.110 | 2.059 | 8 |
| 143 | 2.433 | 1.776 | 8 |
| 144 | 2.812 | 1.319 | 8 |
| 145 | 3.400 | 1.222 | 8 |
| 146 | 2.198 | 1.291 | 8 |
| 147 | 2.977 | 0.992 | 8 |
| 148 | 2.890 | 0.761 | 8 |
| 149 | 3.214 | 1.228 | 8 |
| 150 | 4.393 | 3.821 | 8 |
| 151 | 3.519 | 2.578 | 8 |
| 152 | 3.824 | 2.649 | 8 |
| 153 | 3.927 | 2.913 | 8 |
| 154 | 2.755 | 2.203 | 8 |
| 155 | 3.665 | 2.489 | 8 |
| 156 | 4.786 | 3.746 | 8 |
| 157 | 4.166 | 1.634 | 8 |
| 158 | 4.750 | 3.829 | 8 |
| 159 | 3.432 | 0.959 | 8 |
| 160 | 4.108 | 2.280 | 8 |
| 161 | 4.041 | 2.254 | 9 |
| 162 | 4.013 | 3.996 | 9 |
| 163 | 2.999 | 0.854 | 9 |
| 164 | 3.275 | 3.061 | 9 |
| 165 | 2.417 | 1.554 | 9 |
| 166 | 2.586 | 0.715 | 9 |
| 167 | 2.662 | 0.692 | 9 |
| 168 | 2.444 | 1.023 | 9 |
| 169 | 2.436 | 1.394 | 9 |
| 170 | 4.327 | 2.171 | 9 |
| 171 | 4.370 | 2.109 | 9 |
| 172 | 3.987 | 3.778 | 9 |
| 173 | 3.441 | 1.494 | 9 |
| 174 | 3.693 | 1.499 | 9 |
| 175 | 4.164 | 2.581 | 9 |
| 176 | 4.315 | 2.104 | 9 |
| 177 | 4.149 | 3.581 | 9 |
| 178 | 1.855 | 0.486 | 9 |
| 179 | 4.550 | 3.640 | 9 |
| 180 | 3.688 | 1.048 | 10 |
| 181 | 2.514 | 1.037 | 10 |
| 182 | 4.079 | 2.350 | 10 |
| 183 | 3.398 | 1.277 | 10 |
| 184 | 1.669 | 1.095 | 10 |
| 185 | 3.109 | 1.082 | 10 |
| 186 | 3.382 | 1.509 | 10 |
| 187 | 3.267 | 1.964 | 10 |
| 188 | 2.486 | 1.844 | 10 |
| 189 | 4.372 | 1.949 | 10 |
| 190 | 4.403 | 2.148 | 10 |
| 191 | 4.362 | 2.239 | 10 |
| 192 | 4.331 | 3.169 | 10 |
| 193 | 3.951 | 2.767 | 10 |
| 194 | 3.908 | 2.379 | 10 |
| 195 | 2.850 | 1.158 | 10 |
| 196 | 3.536 | 1.867 | 10 |
| 197 | 4.319 | 3.565 | 10 |
| 198 | 4.216 | 3.209 | 10 |
| 199 | 3.274 | 1.262 | 10 |
| 200 | 4.623 | 1.730 | 10 |
| 201 | 4.596 | 4.904 | 10 |
| 202 | 4.105 | 2.989 | 10 |
| 203 | 4.197 | 3.251 | 10 |
| 204 | 2.284 | 1.472 | 11 |
| 205 | 3.824 | 2.717 | 11 |
| 206 | 4.250 | 2.115 | 11 |
| 207 | 4.243 | 1.340 | 11 |
| 208 | 4.281 | 4.107 | 11 |
| 209 | 3.662 | 2.478 | 11 |
| 210 | 4.821 | 4.417 | 11 |
| 211 | 3.014 | 0.938 | 11 |
| 212 | 4.190 | 1.663 | 11 |
| 213 | 3.513 | 1.226 | 11 |
| 214 | 4.448 | 1.944 | 11 |
| 215 | 2.975 | 1.278 | 12 |
| 216 | 3.835 | 1.123 | 12 |
| 217 | 2.448 | 2.107 | 12 |
| 218 | 3.807 | 2.554 | 12 |
| 219 | 3.677 | 1.346 | 12 |
| 220 | 4.769 | 2.229 | 12 |
| 221 | 3.904 | 2.308 | 12 |
| 222 | 3.622 | 1.324 | 12 |
| 223 | 3.849 | 2.122 | 12 |
| 224 | 4.213 | 3.557 | 12 |
| 225 | 3.399 | 1.591 | 13 |
| 226 | 3.417 | 0.882 | 13 |
| 227 | 3.254 | 1.300 | 13 |
| 228 | 3.690 | 2.329 | 13 |
| 229 | 3.851 | 3.378 | 13 |
| 230 | 4.008 | 3.182 | 13 |
| 231 | 4.498 | 3.493 | 13 |
| 232 | 4.970 | 4.461 | 13 |
| 233 | 4.026 | 3.468 | 14 |
| 234 | 4.022 | 3.711 | 14 |
| 235 | 4.041 | 2.318 | 14 |
| 236 | 3.903 | 2.968 | 14 |
| 237 | 4.320 | 1.619 | 14 |
| 238 | 4.403 | 4.012 | 14 |
| 239 | 3.903 | 0.970 | 14 |
| 240 | 3.513 | 2.069 | 15 |
| 241 | 3.630 | 2.138 | 15 |
| 242 | 5.283 | 3.152 | 15 |
| 243 | 4.952 | 1.983 | 15 |
| 244 | 3.983 | 1.388 | 16 |
| 245 | 5.055 | 2.166 | 16 |
| 246 | 4.358 | 2.123 | 16 |
| 247 | 5.273 | 4.295 | 16 |
| 248 | 4.079 | 4.155 | 17 |
| 249 | 5.232 | 4.241 | 17 |
| 250 | 4.890 | 2.715 | 20 |
| 251 | 2.439 | 0.721 | 3 |
| 252 | 1.431 | 1.106 | 3 |
| 253 | 3.637 | 1.170 | 3 |
| 254 | 4.471 | 3.123 | 3 |
| 255 | 1.769 | 0.298 | 3 |
| 256 | 4.418 | 2.595 | 3 |
| 257 | 2.009 | 0.290 | 3 |
| 258 | 4.422 | 3.232 | 4 |
| 259 | 2.220 | 0.673 | 4 |
| 260 | 2.388 | 1.959 | 4 |
| 261 | 3.761 | 1.888 | 4 |
| 262 | 4.398 | 2.867 | 5 |
| 263 | 2.422 | 1.075 | 5 |
| 264 | 3.139 | 1.798 | 5 |
| 265 | 4.867 | 3.238 | 5 |
| 266 | 3.018 | 0.108 | 5 |
| 267 | 4.586 | 3.098 | 5 |
| 268 | 4.518 | 2.737 | 5 |
| 269 | 3.194 | 0.701 | 5 |
| 270 | 2.562 | 1.563 | 5 |
| 271 | 4.318 | 1.529 | 5 |
| 272 | 4.501 | 3.313 | 5 |
| 273 | 2.498 | 1.625 | 5 |
| 274 | 3.018 | 1.061 | 5 |
| 275 | 3.871 | 1.343 | 5 |
| 276 | 3.316 | 0.915 | 6 |
| 277 | 3.363 | 1.559 | 6 |
| 278 | 4.119 | 3.232 | 6 |
| 279 | 4.454 | 3.232 | 6 |
| 280 | 3.325 | 0.667 | 6 |
| 281 | 2.892 | 1.474 | 6 |
| 282 | 4.357 | 2.287 | 6 |
| 283 | 4.578 | 3.319 | 6 |
| 284 | 4.896 | 3.270 | 6 |
| 285 | 4.533 | 1.816 | 6 |
| 286 | 3.579 | 2.734 | 6 |
| 287 | 2.709 | 3.385 | 6 |
| 288 | 4.286 | 2.723 | 6 |
| 289 | 3.619 | 0.683 | 7 |
| 290 | 3.725 | 3.406 | 7 |
| 291 | 3.681 | 1.879 | 7 |
| 292 | 3.522 | 2.136 | 7 |
| 293 | 5.272 | 3.260 | 7 |
| 294 | 4.676 | 3.114 | 7 |
| 295 | 3.798 | 3.232 | 7 |
| 296 | 4.146 | 1.113 | 7 |
| 297 | 4.330 | 1.795 | 7 |
| 298 | 3.331 | 1.211 | 7 |
| 299 | 4.346 | 3.185 | 7 |
| 300 | 3.445 | 3.313 | 7 |
| 301 | 4.465 | 3.313 | 7 |
| 302 | 2.814 | 2.360 | 7 |
| 303 | 4.769 | 3.313 | 7 |
| 304 | 2.828 | 1.834 | 7 |
| 305 | 4.903 | 3.485 | 7 |
| 306 | 4.903 | 1.847 | 7 |
| 307 | 3.220 | 1.320 | 7 |
| 308 | 3.960 | 2.478 | 7 |
| 309 | 4.244 | 1.811 | 7 |
| 310 | 3.405 | 1.646 | 7 |
| 311 | 4.419 | 1.567 | 8 |
| 312 | 5.272 | 2.713 | 8 |
| 313 | 5.272 | 2.936 | 8 |
| 314 | 4.663 | 3.101 | 8 |
| 315 | 3.760 | 1.300 | 8 |
| 316 | 4.892 | 2.976 | 8 |
| 317 | 3.988 | 1.243 | 8 |
| 318 | 4.867 | 2.987 | 8 |
| 319 | 4.276 | 2.762 | 8 |
| 320 | 4.376 | 1.993 | 8 |
| 321 | 4.291 | 2.782 | 8 |
| 322 | 3.139 | 2.936 | 8 |
| 323 | 4.126 | 2.644 | 8 |
| 324 | 3.896 | 3.260 | 8 |
| 325 | 4.582 | 2.910 | 8 |
| 326 | 4.168 | 2.468 | 8 |
| 327 | 3.507 | 2.219 | 8 |
| 328 | 3.856 | 1.353 | 8 |
| 329 | 3.822 | 2.815 | 8 |
| 330 | 4.024 | 3.485 | 8 |
| 331 | 3.412 | 3.343 | 8 |
| 332 | 4.190 | 3.385 | 8 |
| 333 | 3.835 | 2.628 | 9 |
| 334 | 3.500 | 3.406 | 9 |
| 335 | 3.297 | 2.527 | 9 |
| 336 | 4.133 | 1.574 | 9 |
| 337 | 4.770 | 2.968 | 9 |
| 338 | 4.192 | 1.884 | 9 |
| 339 | 4.052 | 2.632 | 9 |
| 340 | 3.444 | 3.232 | 9 |
| 341 | 4.280 | 3.232 | 9 |
| 342 | 4.277 | 2.669 | 9 |
| 343 | 3.957 | 1.119 | 9 |
| 344 | 3.746 | 1.491 | 9 |
| 345 | 2.165 | 0.579 | 9 |
| 346 | 3.643 | 3.010 | 9 |
| 347 | 3.354 | 2.926 | 9 |
| 348 | 4.672 | 3.348 | 9 |
| 349 | 4.710 | 2.358 | 9 |
| 350 | 4.801 | 3.485 | 9 |
| 351 | 4.575 | 2.446 | 9 |
| 352 | 3.643 | 1.310 | 9 |
| 353 | 4.456 | 2.850 | 9 |
| 354 | 5.222 | 2.986 | 10 |
| 355 | 3.456 | 3.071 | 10 |
| 356 | 4.552 | 2.720 | 10 |
| 357 | 4.593 | 3.406 | 10 |
| 358 | 4.850 | 3.406 | 10 |
| 359 | 4.922 | 3.406 | 10 |
| 360 | 4.816 | 3.406 | 10 |
| 361 | 5.177 | 3.232 | 10 |
| 362 | 4.516 | 3.327 | 10 |
| 363 | 5.026 | 2.744 | 10 |
| 364 | 3.454 | 1.612 | 10 |
| 365 | 4.899 | 3.308 | 10 |
| 366 | 4.220 | 1.159 | 10 |
| 367 | 4.114 | 2.442 | 10 |
| 368 | 4.867 | 3.232 | 10 |
| 369 | 4.643 | 3.198 | 10 |
| 370 | 4.867 | 3.232 | 10 |
| 371 | 3.180 | 0.571 | 10 |
| 372 | 4.508 | 3.242 | 10 |
| 373 | 3.493 | 2.467 | 10 |
| 374 | 4.508 | 2.750 | 10 |
| 375 | 3.465 | 1.797 | 10 |
| 376 | 3.325 | 3.313 | 10 |
| 377 | 4.720 | 3.313 | 10 |
| 378 | 4.632 | 3.313 | 10 |
| 379 | 4.114 | 2.717 | 10 |
| 380 | 4.540 | 2.987 | 10 |
| 381 | 4.185 | 3.264 | 10 |
| 382 | 4.903 | 2.883 | 10 |
| 383 | 4.724 | 3.485 | 10 |
| 384 | 3.450 | 2.901 | 10 |
| 385 | 4.019 | 3.407 | 10 |
| 386 | 3.328 | 3.054 | 10 |
| 387 | 3.892 | 3.124 | 10 |
| 388 | 4.884 | 3.485 | 10 |
| 389 | 4.357 | 3.485 | 10 |
| 390 | 3.899 | 2.192 | 11 |
| 391 | 5.203 | 3.406 | 11 |
| 392 | 5.222 | 3.406 | 11 |
| 393 | 5.222 | 3.287 | 11 |
| 394 | 5.092 | 3.406 | 11 |
| 395 | 4.745 | 1.445 | 11 |
| 396 | 5.054 | 3.345 | 11 |
| 397 | 3.765 | 2.508 | 11 |
| 398 | 4.401 | 3.364 | 11 |
| 399 | 4.801 | 3.232 | 11 |
| 400 | 4.499 | 3.122 | 11 |
| 401 | 3.816 | 3.055 | 11 |
| 402 | 4.912 | 3.232 | 11 |
| 403 | 3.331 | 3.242 | 11 |
| 404 | 2.981 | 0.492 | 11 |
| 405 | 4.240 | 3.074 | 11 |
| 406 | 3.302 | 1.656 | 11 |
| 407 | 4.391 | 3.242 | 11 |
| 408 | 3.652 | 3.242 | 11 |
| 409 | 3.733 | 3.313 | 11 |
| 410 | 4.084 | 2.401 | 11 |
| 411 | 4.486 | 3.485 | 11 |
| 412 | 4.764 | 3.240 | 11 |
| 413 | 4.122 | 3.485 | 11 |
| 414 | 4.274 | 2.232 | 12 |
| 415 | 4.873 | 3.406 | 12 |
| 416 | 5.196 | 3.406 | 12 |
| 417 | 4.930 | 3.406 | 12 |
| 418 | 5.065 | 3.406 | 12 |
| 419 | 3.359 | 1.554 | 12 |
| 420 | 4.922 | 2.449 | 12 |
| 421 | 4.463 | 2.898 | 12 |
| 422 | 2.917 | 1.051 | 12 |
| 423 | 4.128 | 2.283 | 12 |
| 424 | 4.649 | 3.232 | 12 |
| 425 | 4.388 | 2.955 | 12 |
| 426 | 4.556 | 3.242 | 12 |
| 427 | 4.508 | 1.358 | 12 |
| 428 | 3.306 | 1.576 | 12 |
| 429 | 3.184 | 1.615 | 12 |
| 430 | 3.769 | 3.282 | 12 |
| 431 | 4.648 | 2.479 | 12 |
| 432 | 4.865 | 3.209 | 12 |
| 433 | 4.903 | 3.485 | 12 |
| 434 | 5.160 | 2.389 | 13 |
| 435 | 5.222 | 3.234 | 13 |
| 436 | 4.970 | 3.121 | 13 |
| 437 | 4.902 | 3.185 | 13 |
| 438 | 5.127 | 3.421 | 13 |
| 439 | 4.634 | 1.771 | 13 |
| 440 | 4.645 | 3.350 | 13 |
| 441 | 4.915 | 2.934 | 13 |
| 442 | 5.045 | 3.338 | 13 |
| 443 | 5.034 | 2.831 | 13 |
| 444 | 4.784 | 1.391 | 13 |
| 445 | 4.900 | 3.232 | 13 |
| 446 | 4.160 | 3.169 | 13 |
| 447 | 3.820 | 2.746 | 13 |
| 448 | 4.508 | 2.268 | 13 |
| 449 | 4.051 | 3.242 | 13 |
| 450 | 3.646 | 3.002 | 13 |
| 451 | 4.645 | 2.371 | 13 |
| 452 | 4.480 | 3.117 | 13 |
| 453 | 4.601 | 2.895 | 13 |
| 454 | 4.656 | 3.313 | 13 |
| 455 | 4.519 | 2.518 | 13 |
| 456 | 3.921 | 1.997 | 13 |
| 457 | 4.900 | 2.841 | 14 |
| 458 | 4.544 | 3.448 | 14 |
| 459 | 4.934 | 3.364 | 14 |
| 460 | 4.114 | 2.492 | 14 |
| 461 | 4.930 | 3.350 | 14 |
| 462 | 5.034 | 3.350 | 14 |
| 463 | 5.078 | 3.350 | 14 |
| 464 | 4.711 | 2.683 | 14 |
| 465 | 4.848 | 2.681 | 14 |
| 466 | 4.867 | 3.232 | 14 |
| 467 | 4.553 | 3.242 | 14 |
| 468 | 4.426 | 1.871 | 14 |
| 469 | 3.959 | 1.970 | 14 |
| 470 | 4.769 | 3.313 | 14 |
| 471 | 4.769 | 3.313 | 14 |
| 472 | 5.222 | 3.406 | 15 |
| 473 | 4.866 | 3.406 | 15 |
| 474 | 5.005 | 2.778 | 15 |
| 475 | 5.112 | 3.350 | 15 |
| 476 | 5.161 | 3.335 | 15 |
| 477 | 5.201 | 3.062 | 15 |
| 478 | 4.508 | 3.214 | 15 |
| 479 | 3.907 | 2.041 | 15 |
| 480 | 4.508 | 3.242 | 15 |
| 481 | 4.388 | 3.242 | 15 |
| 482 | 4.481 | 3.313 | 15 |
| 483 | 4.691 | 3.313 | 15 |
| 484 | 4.769 | 3.086 | 15 |
| 485 | 4.653 | 3.313 | 15 |
| 486 | 4.817 | 3.232 | 15 |
| 487 | 5.212 | 1.943 | 16 |
| 488 | 5.272 | 3.350 | 16 |
| 489 | 5.054 | 2.430 | 16 |
| 490 | 4.195 | 1.650 | 16 |
| 491 | 4.876 | 3.232 | 16 |
| 492 | 4.517 | 3.341 | 16 |
| 493 | 4.548 | 3.313 | 16 |
| 494 | 5.157 | 3.406 | 17 |
| 495 | 4.960 | 3.169 | 17 |
| 496 | 4.867 | 3.232 | 17 |
| 497 | 3.914 | 2.508 | 17 |
| 498 | 4.821 | 3.313 | 17 |
| 499 | 4.809 | 3.406 | 18 |
| 500 | 4.914 | 3.350 | 18 |
| 501 | 3.898 | 2.176 | 18 |
| 502 | 5.022 | 3.406 | 19 |
| 503 | 5.200 | 3.350 | 19 |
| 504 | 4.508 | 3.242 | 19 |
| 505 | 4.036 | 3.406 | 20 |
| 506 | 5.102 | 3.350 | 20 |
| 507 | 1.197 | 0.576 | 3 |
| 508 | 2.777 | 0.723 | 8 |
| 509 | 3.162 | 0.954 | 10 |
| 510 | 3.969 | 3.024 | 15 |
| 511 | 2.450 | 1.009 | 10 |
| 512 | 3.830 | 1.590 | 12 |
| 513 | 0.320 | 0.165 | 2 |
| 514 | 2.401 | 0.819 | 8 |
| 515 | 0.835 | 0.382 | 3 |
| 516 | 3.820 | 1.796 | 7 |
| 517 | 4.648 | 3.737 | 14 |
| 518 | 1.156 | 0.345 | 4 |
| 519 | 0.631 | 0.212 | 2 |
| 520 | 4.648 | 3.731 | 13 |
| 521 | 2.690 | 1.259 | 9 |
| 522 | 3.808 | 1.727 | 7 |
| 523 | 2.083 | 1.979 | 7 |
| 524 | 1.833 | 0.567 | 8 |
| 525 | 1.241 | 0.445 | 2 |
| 526 | 2.594 | 1.868 | 4 |
| 527 | 2.323 | 1.085 | 9 |
| 528 | 1.815 | 0.557 | 8 |
| 529 | 0.589 | 0.360 | 3 |
| 530 | 4.375 | 3.068 | 15 |
| 531 | 2.528 | 0.978 | 10 |
| 532 | 1.774 | 0.803 | 5 |
| 533 | 0.176 | 0.105 | 3 |
| 534 | 0.432 | 0.345 | 5 |
| 535 | 1.860 | 0.794 | 3 |
| 536 | 3.059 | 1.546 | 9 |
| 537 | 1.154 | 0.700 | 2 |
| 538 | 4.611 | 3.570 | 16 |
| 539 | 0.577 | 0.173 | 2 |
| 540 | 1.182 | 0.170 | 3 |
| 541 | 0.181 | 0.142 | 1 |
| 542 | 1.193 | 0.429 | 2 |
| 543 | 1.579 | 1.807 | 5 |
| 544 | 1.255 | 0.611 | 7 |
| 545 | 1.138 | 0.339 | 7 |
| 546 | 0.209 | 0.124 | 0 |
| 547 | 0.572 | 0.320 | 2 |
| 548 | 0.630 | 0.369 | 3 |
| 549 | 3.586 | 1.643 | 11 |
| 550 | 1.125 | 0.637 | 3 |
| 551 | 3.089 | 2.221 | 12 |
| 552 | 2.679 | 1.927 | 11 |
| 553 | 0.245 | 0.157 | 2 |
| 554 | 2.842 | 1.497 | 12 |
| 555 | 2.599 | 2.586 | 9 |
| 556 | 2.154 | 0.763 | 8 |
| 557 | 3.817 | 4.218 | 16 |
| 558 | 2.317 | 1.635 | 11 |
| 559 | 2.595 | 1.832 | 11 |
| 560 | 2.734 | 1.773 | 12 |
| 561 | 3.273 | 3.769 | 9 |
| 562 | 1.990 | 0.370 | 7 |
| 563 | 1.539 | 0.573 | 7 |
| 564 | 2.511 | 1.433 | 11 |
| 565 | 2.239 | 1.106 | 8 |
| 566 | 3.860 | 4.221 | 15 |
| 567 | 2.975 | 1.015 | 10 |
| 568 | 2.512 | 1.021 | 8 |
| 569 | 3.728 | 3.671 | 14 |
| 570 | 2.060 | 2.216 | 5 |
| 571 | 3.357 | 2.988 | 11 |
| 572 | 2.166 | 1.272 | 7 |
| 573 | 3.899 | 4.715 | 11 |
| 574 | 2.876 | 2.586 | 10 |
| 575 | 2.832 | 1.646 | 7 |
| 576 | 2.541 | 1.217 | 12 |
| 577 | 3.540 | 3.047 | 14 |
| 578 | 3.041 | 3.610 | 13 |
| 579 | 1.853 | 1.152 | 8 |
| 580 | 3.267 | 2.534 | 11 |
| 581 | 3.185 | 2.635 | 12 |
| 582 | 3.098 | 2.572 | 11 |
