# Supplementary material for: Development of a New Limiting-Antigen Avidity Dot Immuno-Gold Filtration Assay for HIV-1 Incidence
Source: PLoS One. 2016 Aug 11;11(8):e0161183. doi: 10.1371/journal.pone.0161183 (PMC4981313; doi:10.1371/journal.pone.0161183)
Supplement: S2 Table — (DOCX) [file pone.0161183.s002.docx]

**S2 Table. BED-CEIA ODn, LAg-Avidity ODn, the gray values and CD4^+^ count of specimens from panel 5.**

| **NO.** | **ODn**  **LAg-Avidity EIA** | **ODn**  **BED-CEIA** | **Gray Value**  **(DIGSSA)** | **CD4 count** |
| --- | --- | --- | --- | --- |
| 1 | 3.500 | 3.406 | 9 | 421 |
| 2 | 4.900 | 2.841 | 14 | 353 |
| 3 | 4.544 | 3.448 | 14 | 191 |
| 4 | 4.274 | 2.232 | 12 | 701 |
| 5 | 5.157 | 3.406 | 17 | No detect |
| 6 | 5.222 | 2.986 | 10 | No detect |
| 7 | 4.934 | 3.364 | 14 | No detect |
| 8 | 5.160 | 2.389 | 13 | No detect |
| 9 | 3.899 | 2.192 | 11 | No detect |
| 10 | 5.203 | 3.406 | 11 | 762 |
| 11 | 5.222 | 3.406 | 11 | 459 |
| 12 | 3.456 | 3.071 | 10 | 668 |
| 13 | 4.552 | 2.720 | 10 | 479 |
| 14 | 4.873 | 3.406 | 12 | 741 |
| 15 | 5.196 | 3.406 | 12 | 684 |
| 16 | 5.222 | 3.234 | 13 | 629 |
| 17 | 4.593 | 3.406 | 10 | 782 |
| 18 | 5.222 | 3.287 | 11 | 266 |
| 19 | 4.970 | 3.121 | 13 | 403 |
| 20 | 3.297 | 2.527 | 9 | 748 |
| 21 | 4.850 | 3.406 | 10 | 624 |
| 22 | 4.922 | 3.406 | 10 | 503 |
| 23 | 4.902 | 3.185 | 13 | 1071 |
| 24 | 4.816 | 3.406 | 10 | 442 |
| 25 | 5.092 | 3.406 | 11 | 1217 |
| 26 | 5.127 | 3.421 | 13 | 573 |
| 27 | 4.745 | 1.445 | 11 | 1050 |
| 28 | 3.619 | 0.683 | 7 | 1051 |
| 29 | 5.177 | 3.232 | 10 | 847 |
| 30 | 4.114 | 2.492 | 14 | 702 |
| 31 | 4.036 | 3.406 | 20 | 1010 |
| 32 | 4.930 | 3.406 | 12 | 317 |
| 33 | 5.054 | 3.345 | 11 | 528 |
| 34 | 4.634 | 1.771 | 13 | 392 |
| 35 | 3.725 | 3.406 | 7 | 595 |
| 36 | 5.222 | 3.406 | 15 | 547 |
| 37 | 4.809 | 3.406 | 18 | 665 |
| 38 | 5.065 | 3.406 | 12 | 460 |
| 39 | 5.022 | 3.406 | 19 | 764 |
| 40 | 5.212 | 1.943 | 16 | 638 |
| 41 | 4.866 | 3.406 | 15 | 314 |
| 42 | 4.133 | 1.574 | 9 | 778 |
| 43 | 5.005 | 2.778 | 15 | 449 |
| 44 | 5.112 | 3.350 | 15 | 581 |
| 45 | 3.681 | 1.879 | 7 | 387 |
| 46 | 3.765 | 2.508 | 11 | 264 |
| 47 | 5.161 | 3.335 | 15 | 948 |
| 48 | 4.401 | 3.364 | 11 | 345 |
| 49 | 4.914 | 3.350 | 18 | 566 |
| 50 | 3.359 | 1.554 | 12 | 347 |
| 51 | 4.645 | 3.350 | 13 | 533 |
| 52 | 4.770 | 2.968 | 9 | 374 |
| 53 | 4.516 | 3.327 | 10 | 332 |
| 54 | 4.930 | 3.350 | 14 | 424 |
| 55 | 2.439 | 0.721 | 3 | 178 |
| 56 | 5.272 | 2.713 | 8 | 899 |
| 57 | 4.915 | 2.934 | 13 | 322 |
| 58 | 5.201 | 3.062 | 15 | 527 |
| 59 | 5.034 | 3.350 | 14 | 359 |
| 60 | 5.272 | 3.350 | 16 | 402 |
| 61 | 5.026 | 2.744 | 10 | 513 |
| 62 | 3.522 | 2.136 | 7 | 178 |
| 63 | 5.054 | 2.430 | 16 | 181 |
| 64 | 4.195 | 1.650 | 16 | 476 |
| 65 | 5.045 | 3.338 | 13 | 904 |
| 66 | 5.078 | 3.350 | 14 | 317 |
| 67 | 3.454 | 1.612 | 10 | 539 |
| 68 | 5.034 | 2.831 | 13 | 495 |
| 69 | 4.899 | 3.308 | 10 | 387 |
| 70 | 3.316 | 0.915 | 6 | 358 |
| 71 | 5.272 | 3.260 | 7 | 690 |
| 72 | 5.272 | 2.936 | 8 | 820 |
| 73 | 5.102 | 3.350 | 20 | 258 |
| 74 | 4.960 | 3.169 | 17 | 450 |
| 75 | 4.922 | 2.449 | 12 | 166 |
| 76 | 5.200 | 3.350 | 19 | 323 |
| 77 | 2.422 | 1.075 | 5 | 361 |
| 78 | 4.784 | 1.391 | 13 | 475 |
| 79 | 3.363 | 1.559 | 6 | 672 |
| 80 | 4.663 | 3.101 | 8 | 516 |
| 81 | 4.220 | 1.159 | 10 | 262 |
| 82 | 4.711 | 2.683 | 14 | 163 |
| 83 | 1.431 | 1.106 | 3 | 559 |
| 84 | 4.848 | 2.681 | 14 | 583 |
| 85 | 4.801 | 3.232 | 11 | 656 |
| 86 | 4.499 | 3.122 | 11 | 192 |
| 87 | 4.900 | 3.232 | 13 | 199 |
| 88 | 4.192 | 1.884 | 9 | 157 |
| 89 | 3.637 | 1.170 | 3 | 476 |
| 90 | 3.760 | 1.300 | 8 | 257 |
| 91 | 4.676 | 3.114 | 7 | 405 |
| 92 | 4.867 | 3.232 | 14 | 98 |
| 93 | 4.463 | 2.898 | 12 | 524 |
| 94 | 4.892 | 2.976 | 8 | 295 |
| 95 | 4.114 | 2.442 | 10 | 389 |
| 96 | 2.917 | 1.051 | 12 | 1002 |
| 97 | 4.128 | 2.283 | 12 | 487 |
| 98 | 4.867 | 3.232 | 17 | 314 |
| 99 | 4.876 | 3.232 | 16 | 483 |
| 100 | 4.649 | 3.232 | 12 | 455 |
| 101 | 3.139 | 1.798 | 5 | 284 |
| 102 | 4.052 | 2.632 | 9 | 821 |
| 103 | 3.988 | 1.243 | 8 | 294 |
| 104 | 4.867 | 3.232 | 10 | 322 |
| 105 | 4.867 | 2.987 | 8 | 665 |
| 106 | 3.444 | 3.232 | 9 | 324 |
| 107 | 4.471 | 3.123 | 3 | 449 |
| 108 | 4.643 | 3.198 | 10 | 542 |
| 109 | 4.276 | 2.762 | 8 | 767 |
| 110 | 4.817 | 3.232 | 15 | 341 |
| 111 | 4.119 | 3.232 | 6 | 493 |
| 112 | 4.422 | 3.232 | 4 | 406 |
| 113 | 4.280 | 3.232 | 9 | 662 |
| 114 | 4.867 | 3.238 | 5 | 790 |
| 115 | 4.867 | 3.232 | 10 | 370 |
| 116 | 3.816 | 3.055 | 11 | 192 |
| 117 | 3.018 | 0.108 | 5 | 474 |
| 118 | 3.798 | 3.232 | 7 | 850 |
| 119 | 4.146 | 1.113 | 7 | 438 |
| 120 | 4.912 | 3.232 | 11 | 606 |
| 121 | 4.277 | 2.669 | 9 | 446 |
| 122 | 3.957 | 1.119 | 9 | 468 |
| 123 | 4.454 | 3.232 | 6 | 507 |
| 124 | 4.586 | 3.098 | 5 | 332 |
| 125 | 4.330 | 1.795 | 7 | 435 |
| 126 | 4.376 | 1.993 | 8 | 477 |
| 127 | 3.331 | 3.242 | 11 | 231 |
| 128 | 4.291 | 2.782 | 8 | 296 |
| 129 | 4.518 | 2.737 | 5 | 175 |
| 130 | 4.553 | 3.242 | 14 | 413 |
| 131 | 3.180 | 0.571 | 10 | 678 |
| 132 | 4.508 | 3.214 | 15 | 267 |
| 133 | 4.160 | 3.169 | 13 | 264 |
| 134 | 4.388 | 2.955 | 12 | 270 |
| 135 | 4.508 | 3.242 | 10 | 254 |
| 136 | 2.981 | 0.492 | 11 | 586 |
| 137 | 4.556 | 3.242 | 12 | 365 |
| 138 | 4.508 | 1.358 | 12 | 365 |
| 139 | 3.820 | 2.746 | 13 | 378 |
| 140 | 4.240 | 3.074 | 11 | 510 |
| 141 | 4.426 | 1.871 | 14 | 309 |
| 142 | 3.907 | 2.041 | 15 | 562 |
| 143 | 3.306 | 1.576 | 12 | 221 |
| 144 | 3.194 | 0.701 | 5 | 110 |
| 145 | 2.220 | 0.673 | 4 | 560 |
| 146 | 3.302 | 1.656 | 11 | 391 |
| 147 | 3.331 | 1.211 | 7 | 334 |
| 148 | 4.508 | 3.242 | 15 | 393 |
| 149 | 4.391 | 3.242 | 11 | 186 |
| 150 | 3.959 | 1.970 | 14 | 115 |
| 151 | 4.508 | 2.268 | 13 | 710 |
| 152 | 4.508 | 3.242 | 19 | 766 |
| 153 | 4.388 | 3.242 | 15 | 591 |
| 154 | 3.898 | 2.176 | 18 | No detect |
| 155 | 3.914 | 2.508 | 17 | 26 |
| 156 | 3.184 | 1.615 | 12 | 671 |
| 157 | 3.746 | 1.491 | 9 | 28 |
| 158 | 3.769 | 3.282 | 12 | 404 |
| 159 | 1.769 | 0.298 | 3 | 1153 |
| 160 | 3.493 | 2.467 | 10 | 587 |
| 161 | 3.652 | 3.242 | 11 | 318 |
| 162 | 4.051 | 3.242 | 13 | 349 |
| 163 | 4.346 | 3.185 | 7 | 914 |
| 164 | 4.508 | 2.750 | 10 | 708 |
| 165 | 3.646 | 3.002 | 13 | 803 |
| 166 | 2.165 | 0.579 | 9 | 763 |
| 167 | 3.445 | 3.313 | 7 | 1508 |
| 168 | 3.325 | 0.667 | 6 | 734 |
| 169 | 4.481 | 3.313 | 15 | 680 |
| 170 | 4.645 | 2.371 | 13 | 891 |
| 171 | 3.465 | 1.797 | 10 | 430 |
| 172 | 4.480 | 3.117 | 13 | 449 |
| 173 | 4.691 | 3.313 | 15 | 639 |
| 174 | 4.821 | 3.313 | 17 | 475 |
| 175 | 4.517 | 3.341 | 16 | 573 |
| 176 | 3.325 | 3.313 | 10 | 459 |
| 177 | 4.548 | 3.313 | 16 | 932 |
| 178 | 3.139 | 2.936 | 8 | 1073 |
| 179 | 4.126 | 2.644 | 8 | 659 |
| 180 | 4.720 | 3.313 | 10 | 317 |
| 181 | 4.632 | 3.313 | 10 | 509 |
| 182 | 4.465 | 3.313 | 7 | 415 |
| 183 | 4.114 | 2.717 | 10 | 792 |
| 184 | 3.643 | 3.010 | 9 | 507 |
| 185 | 3.733 | 3.313 | 11 | 490 |
| 186 | 2.892 | 1.474 | 6 | 719 |
| 187 | 3.354 | 2.926 | 9 | 922 |
| 188 | 2.814 | 2.360 | 7 | 941 |
| 189 | 4.357 | 2.287 | 6 | 601 |
| 190 | 4.769 | 3.313 | 14 | 697 |
| 191 | 3.896 | 3.260 | 8 | 426 |
| 192 | 4.769 | 3.086 | 15 | 333 |
| 193 | 2.562 | 1.563 | 5 | 1046 |
| 194 | 4.540 | 2.987 | 10 | 343 |
| 195 | 4.084 | 2.401 | 11 | 1339 |
| 196 | 4.769 | 3.313 | 14 | 479 |
| 197 | 4.672 | 3.348 | 9 | 226 |
| 198 | 4.318 | 1.529 | 5 | 228 |
| 199 | 4.418 | 2.595 | 3 | 188 |
| 200 | 4.501 | 3.313 | 5 | 584 |
| 201 | 4.710 | 2.358 | 9 | 191 |
| 202 | 4.769 | 3.313 | 7 | 390 |
| 203 | 4.601 | 2.895 | 13 | 236 |
| 204 | 4.656 | 3.313 | 13 | 501 |
| 205 | 4.653 | 3.313 | 15 | 290 |
| 206 | 2.828 | 1.834 | 7 | 495 |
| 207 | 4.185 | 3.264 | 10 | 354 |
| 208 | 4.903 | 2.883 | 10 | 323 |
| 209 | 4.648 | 2.479 | 12 | 332 |
| 210 | 4.519 | 2.518 | 13 | 72 |
| 211 | 4.486 | 3.485 | 11 | 347 |
| 212 | 4.578 | 3.319 | 6 | 580 |
| 213 | 4.903 | 3.485 | 7 | 822 |
| 214 | 4.903 | 1.847 | 7 | 379 |
| 215 | 4.724 | 3.485 | 10 | 407 |
| 216 | 3.450 | 2.901 | 10 | 823 |
| 217 | 3.220 | 1.320 | 7 | 793 |
| 218 | 2.498 | 1.625 | 5 | 494 |
| 219 | 3.921 | 1.997 | 13 | 394 |
| 220 | 4.019 | 3.407 | 10 | 507 |
| 221 | 3.328 | 3.054 | 10 | 328 |
| 222 | 4.896 | 3.270 | 6 | 584 |
| 223 | 4.582 | 2.910 | 8 | 395 |
| 224 | 4.801 | 3.485 | 9 | 195 |
| 225 | 4.533 | 1.816 | 6 | 578 |
| 226 | 2.388 | 1.959 | 4 | 559 |
| 227 | 4.419 | 1.567 | 8 | 846 |
| 228 | 4.168 | 2.468 | 8 | 427 |
| 229 | 4.575 | 2.446 | 9 | 340 |
| 230 | 3.643 | 1.310 | 9 | 920 |
| 231 | 4.764 | 3.240 | 11 | 338 |
| 232 | 3.018 | 1.061 | 5 | 260 |
| 233 | 3.507 | 2.219 | 8 | 118 |
| 234 | 3.892 | 3.124 | 10 | 561 |
| 235 | 4.456 | 2.850 | 9 | 106 |
| 236 | 4.865 | 3.209 | 12 | 1067 |
| 237 | 3.856 | 1.353 | 8 | 602 |
| 238 | 3.871 | 1.343 | 5 | 217 |
| 239 | 3.822 | 2.815 | 8 | 553 |
| 240 | 4.903 | 3.485 | 12 | 623 |
| 241 | 4.122 | 3.485 | 11 | 526 |
| 242 | 3.761 | 1.888 | 4 | 718 |
| 243 | 4.398 | 2.867 | 5 | 433 |
| 244 | 3.960 | 2.478 | 7 | 284 |
| 245 | 4.244 | 1.811 | 7 | 294 |
| 246 | 3.579 | 2.734 | 6 | 262 |
| 247 | 4.024 | 3.485 | 8 | 81 |
| 248 | 4.884 | 3.485 | 10 | 905 |
| 249 | 4.357 | 3.485 | 10 | 450 |
| 250 | 3.405 | 1.646 | 7 | 993 |
| 251 | 2.709 | 3.385 | 6 | 374 |
| 252 | 3.412 | 3.343 | 8 | 419 |
| 253 | 4.286 | 2.723 | 6 | 317 |
| 254 | 4.190 | 3.385 | 8 | 489 |
| 255 | 3.835 | 2.628 | 9 | 230 |
| 256 | 2.009 | 0.290 | 3 | 509 |
